# Predictors of outcome after CABG in the South-Asian community: a propensity matched analysis

**DOI:** 10.1177/02676591211037577

**Published:** 2021-08-07

**Authors:** Mohammad Yousuf Salmasi, Ramanish Ravishankar, Yusuf Abdullahi, Philip Hartley, Panagiotis G Kyriazis, Thanos Athanasiou, Prakash Punjabi

**Affiliations:** 1 Department of Surgery, Imperial College London, London, UK; 2 Department of Cardiac Surgery, Imperial College Healthcare NHS Trust, London, UK

**Keywords:** coronary artery bypass, ethnicity, Asian, creatinine

## Abstract

**Background:**

Ethnicity is not incorporated into standardized pre-operative risk-stratification tools for cardiac surgery. This study compared short-term outcomes following coronary artery bypass graft (CABG) surgery in South Asian and non-Asian patients.

**Methods:**

Consecutive patients undergoing isolated CABG surgery via sternotomy between the years 2011 and 2019 were retrospectively analyzed. Initially, 1957 patients were identified (799 South-Asian, 40.8%). The patient groups were then propensity matched according to 10 relevant pre-operative covariates (age, body mass index, pulmonary disease, renal failure, smoking, diabetes, ventricular function, renal failure): 675 non-Asian patients were matched against 675 Asian patients.

**Results:**

Operative mortality was 1.77% and similar between the two groups (p = 0.447). Multivariate regression analysis found predictors of operative mortality to be pre-operative serum creatinine, age, left ventricular (LV) impairment, and extent of coronary disease. The effect of creatinine on mortality was selective for South-Asian patients (p = 0.015). LV impairment was a predictor of mortality in non-Asian patients, however this effect did not exist in South-Asian patients. Predictors of short-term complications (composite of death, stroke, reoperation, hemofiltration, and pneumonia) were age and creatinine (coefficient 0.002, 95% CI 0.0004–0.004, p = 0.019) in the overall cohort. Subgroup analysis found age to remain a selective negative predictor of complications in South-Asian patients. Cox regression analysis found creatinine, age, and LVEF to influence 10-year survival, whilst ethnicity was not a predictor.

**Conclusion:**

This study highlights the cumulative risk associated with ethnicity and renal disease in predicting short-term outcomes following CABG. This warrants further investigations in larger populations, thus guiding pre-operative risk-stratification.

## Introduction

Coronary artery bypass graft (CABG) surgery is an extremely common method of myocardial revascularization.^1^ Given the ethnic diversity among the population suffering coronary artery disease,^2^ and the known variations in cardiovascular phenotypes amongst different ethnic populations, the influence of this variable on operative risk-stratification and management is important to consider.

Ethnicity has been shown to influence hospital mortality following CABG, with a trend toward increased mortality in Asian patients compared with Caucasian patients.^3^ One school of thought is that the reduced number of referrals in ethnic minorities for coronary revascularization leads these patients to present at more advanced stages of disease and have a greater operative risk than Caucasian patients.^4,5^ In addition, the prevalence of hypertension, diabetes, and hypercholesterolemia are increased in the Asian population,^6,7^ which can contribute to cardiovascular disease burden, coronary targets, and fitness for recovery, thus affecting the risk of surgery as a whole.

Of topical importance, complex variations between ethnicities has been further exposed during the recent coronavirus disease 2019 (COVID-19) pandemic, which has demonstrated both the susceptibility of black and minority ethnic groups to morbidity, as well as the increased risk of mortality.^8^

Despite its effect on procedural mortality, there is no clear consensus on the influence of South Asian ethnicity on multiple outcomes following CABG. As such, ethnicity is not currently incorporated into standardized pre-operative risk-stratification tools.^5,9,10^ The aim of this study is to explore the outcomes in a large cohort of patients undergoing CABG surgery, comparing groups of South-Asian and Non-Asian patients using propensity matched analysis.

## Methods

The study was granted an ethical waiver by the Joint Research and Compliance Office (Imperial College NHS Trust). Perioperative data was retrospectively analyzed from a prospectively collated database at a single cardiothoracic institution between 2011 and 2019. The cardiothoracic service has a designated patient liaison team dedicated to informing patients of treatment progress and research involvement. Patients undergoing treatment consented to data being used anonymously for data repository and research. Patients or the public were not involved in the design or conduct of this research study.

### Operative technique

All patients underwent median sternotomy for operative access. The technique for cardiopulmonary bypass (CPB) was similar in all patients: this was achieved via central cannulation (of the ascending aorta and right atrium). All patients were cooled to a mild hypothermia at a temperature of 32°C–34°C. Following application of the aortic cross clamp, antegrade cold blood cardioplegia was infused via the aortic root, and intermittently thereafter via the root and anastomosed vein grafts. This cardioprotection strategy was used for all patients.

Patients received an internal mammary artery (IMA) to the left anterior descending (LAD), typically the left mammary (LIMA) and saphenous vein grafts (SVG) to the other coronary targets. All patients received a minimum of two bypass grafts. Neither bilateral IMA nor radial artery conduits were used.

Post-operative management followed a structured protocol for all patients, initially in an intensive care unit (ICU) for the first 24 hours followed by gradual step-down upon patient recovery from surgery. Extubation was targeted for 6 hours where possible and chest drains for removal in the first 24–48 hours. Post-operative complications, were identified according to published protocols^11^ and managed according to best practice.

### Data collection

The cardiac surgical database is locally managed and centrally overseen at a national level, following national guidelines for minimal perioperative data collection, including pre-operative co-variates, detailed operative characteristics, and short-term post-operative outcomes.

Patient ethnicity was obtained from routine hospital records (collected on patient admission) which distinguish South-Asian patients (Indian, Pakistani, Bangladeshi) from other ethnicities.

### Propensity-matching procedure

In order to identify a matched group of Non-Asian versus South-Asian patients, the preoperative and intraoperative covariates associated (p < 0.1) with South-Asian patients were identified. These univariate factors were entered into a multivariable stepwise forward logistic regression analysis to identify the independent factors associated with South-Asians. Based on this model, each patient received a score for “propensity.”

Patients in the South-Asian group were divided into deciles according to the distribution of the propensity score. For each decile, the interval of propensity scores was identified. The propensity-matched control group was created by randomly selecting, per each decile, an equal number of patients from the Non-Asian group, with an equal distribution and range of propensity score. The two groups of 675 patients were checked for homogeneity at the end of the procedure.

### Statistical analysis

Results were analyzed and presented as means and standard deviations. Pre-operative covariates were assessed for normal distribution using the Shapiro-Wilk test. Between group characteristics were assessed for statistical differences using the Student *T* test or Wilcoxon Rank Test for non-parametric variables. Multivariate logistic regression models were constructed to assess the influence of a variety of covariates on short term outcomes. Linear regression was used to assess the influence of covariates on parametric outcomes, namely hospital stay. Adjusted odds ratio with 95% confidence interval (CI) of binary outcomes were calculated.

Crude survival curves were estimated using the nonparametric Kaplan-Meier method, and log rank tests were used to compare the survival distribution among groups. Cox proportional hazard regression was conducted to calculate the adjusted hazards ratio with 95% CI. Stepwise selection was performed using age, sex, COPD, Euroscore, NYHA class, and LV function in the model. Statistical analyses were conducted using the Stata 13.0 software (Stata Corp., College Station, TX, USA).

## Results

In the study period, 1957 patients underwent CABG surgery (799 South-Asian, 40.8%). The patient groups were propensity matched according to 10 relevant pre-operative covariates (Age, gender, BMI, COPD, renal failure, smoking, diabetes, LVEF, peripheral vascular disease, operating surgeon): 675 non-Asian patients were matched against 675 South-Asian patients.

### Pre-operative covariates

For the overall study cohort, (*n* = 1158 vs 799 Non-Asian vs South-Asian), between group differences were significant for a number of covariates, including (Non-Asian vs South-Asian): age (mean 63.0 vs 66.3, p < 0.001), diabetes (33.4% vs 36.7%, p < 0.001), smoking history (67.9% vs 53.4%, p < 0.001), renal failure (2.2% vs 4.0%, p = 0.022), chronic obstructive pulmonary disease (COPD) (9.3% vs 6.9%, p = 0.049), and extent of coronary disease (Supplemental Appendix). The South-Asian cohort was older in age, with a higher incidence of diabetes and renal failure as well a higher proportion of patients with at least three-vessel coronary disease. On the hand, the non-Asian cohort had a significantly higher number of smokers and patients with COPD.

Through propensity matching (*n* = 675 vs 675), the two cohorts became comparable for the aforementioned covariates (non-Asian vs South-Asian): age (65.1 vs 63.0, p = 0.265), diabetes (52.1% vs 60.7%, p = 0.145), renal failure (2.7% vs 3.4%, p = 0.190), COPD (9.9% vs 8.1%, p = 0.129), and extent of coronary vessel disease (p = 0.353). The propensity matched cohorts were also comparable with other pre-operative variables (Table 1).

**Table 1.** Summary statistics of propensity matched cohort.

Covariate	Non-Asian (*n* = 675) (%)	South Asian (*n* = 675) (%)	p-Value
Age	65.1 ± 11.4	63.0 ± 10.7	0.265
Female	156 (23.1)	172 (25.5)	0.139
NYHA class	2.22 ± 0.73	2.28 ± 0.73	0.155
NYHA III–IV	231 (34.2)	273 (40.4)	0.240
Diabetes	352 (52.1)	410 (60.7)	0.145
Hypertension	577 (85.5)	544 (80.6)	0.361
Smoking history	414 (61.3)	380 (56.3)	0.612
Creatinine	99.8 ± 74.1	106.6 ± 92.9	0.382
Renal failure	18 (2.7)	23 (3.4)	0.190
COPD	67 (9.9)	55 (8.1)	0.129
Neurological dysfunction	26 (3.9)	15 (2.2)	0.100
Peripheral vascular disease	59 (8.7)	52 (7.7)	0.583
Extent of coronary disease			0.353
1	82 (12.1)	62 (9.2)	
2	130 (19.2)	110 (16.3)	
3 or more	463 (68.6)	503 (74.5)	
LVEF	52.4 ± 8.7	52.7 ± 7.8	0.510
LV impairment	151 (22.4)	177 (26.2)	0.919
Height	169.7 ± 9.7	166.9 ± 9.4	0.382
Weight	81.3 ± 16.7	77.2 ± 15.1	0.430
BMI	28.1 ± 5.0	27.6 ± 4.9	0.678
Cross clamp time	49.4 ± 34.6	44.1 ± 26.6	0.230
CPB time	86.5 ± 55.3	82.6 ± 33.1	0.140

LVEF: left ventricular ejection fraction; COPD: chronic obstructive pulmonary disease; BMI: body mass index; NYHA: New York Heart Association; CPB: cardiopulmonary bypass.

### Short-term outcomes

Outcomes were compared for propensity matched cohorts. Operative mortality was 1.77% and, whilst it was 0.9% higher in South-Asian patients, this difference was non-significant (1.5% vs 2.4%, p = 0.446). The incidence of new onset neurological events in both groups was also similar (0.7% vs 0.3%, p = 0.371) and this trend was seen for surgical site infection (SSI) incidence (1.9% vs 1.5%, p = 0.851), hemofiltration (2.2% vs 2.2%, p = 0.63), and the use of intra-aortic balloon pumps (IABP) (0.9% vs 0.4%, p = 0.452). A similar result was also seen with the reoperation rates (4.4% vs 4.1%, p = 0.501).

### Multivariate analysis

A multivariate model was constructed using propensity matched data to sequentially assess the effect of potential pre-operative covariates on outcomes of interest (Table 2).

**Table 2.** Multivariate regression analysis, predictors of operative mortality.

Covariate	Odds ratio	95% CI	Standard error	p-Value
Whole cohort				
Age	1.11	1.05–1.17	0.031	**<0.0001**
South-Asian ethnicity	1.54	0.60–3.96	0.74	0.370
Female	1.86	0.82–4.22	0.78	0.134
Smoking	0.76	0.36–1.64	0.30	0.490
NYHA class	1.12	0.61–2.05	0.35	0.714
LV impairment	2.62	1.19–5.75	1.05	**0.017**
Creatinine	1.06	1.00 - 1.12	0.0012	**0.001**
No. coronary vessels diseased	1.98	1.23–3.19	0.48	**0.005**
Cross clamp time	1.01	0.99 - 1.02	0.0073	0.228
South-Asian cohort only				
Age	1.10	1.04–1.07	0.033	**0.002**
Creatinine	1.03	1.01–1.06	0.0014	**0.015**
LV impairment	1.61	0.55–4.69	0.88	0.385
No. coronary vessels diseased	1.31	0.77–2.24	0.36	0.324
Non-Asian cohort only				
Age	1.08	1.01–1.16	0.038	**0.031**
Creatinine	1.01	0.96–1.07	0.0028	0.492
LV impairment	5.41	1.51–19.45	3.53	**0.010**
No. coronary vessels diseased	1.42	0.72–2.81	0.49	0.308

Results from logistic regression.

Bold type denotes statistical significance.

The significant predictors of operative mortality were age (OR 1.11, 95% CI 1.05–1.17, p < 0.0001), preoperative creatinine (OR 1.06, 95% CI 1.00–1.12, p = 0.001), left ventricular impairment (OR 2.62, 95% CI 1.19–5.57, p = 0.017), and the extent of coronary vessel disease (OR 1.98, 95% CI 1.23–3.19, p = 0.005). South Asian ethnicity (p = 0.37), female gender (p = 0.134), smoking (p = 0.49), NYHA class (p = 0.714), and cross clamp time (p = 0.228) were all found to be non-significant predictors of mortality.

**Figure 1.**
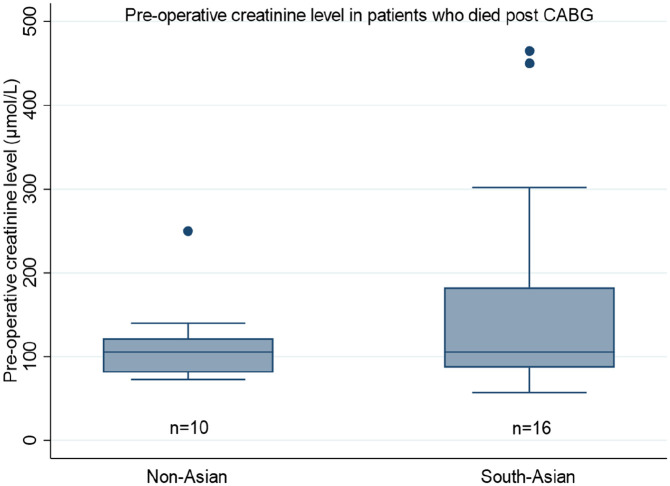
Pre-operative serum creatine levels amongst patients who died following CABG surgery (*n* = 26).

**Figure 2.**
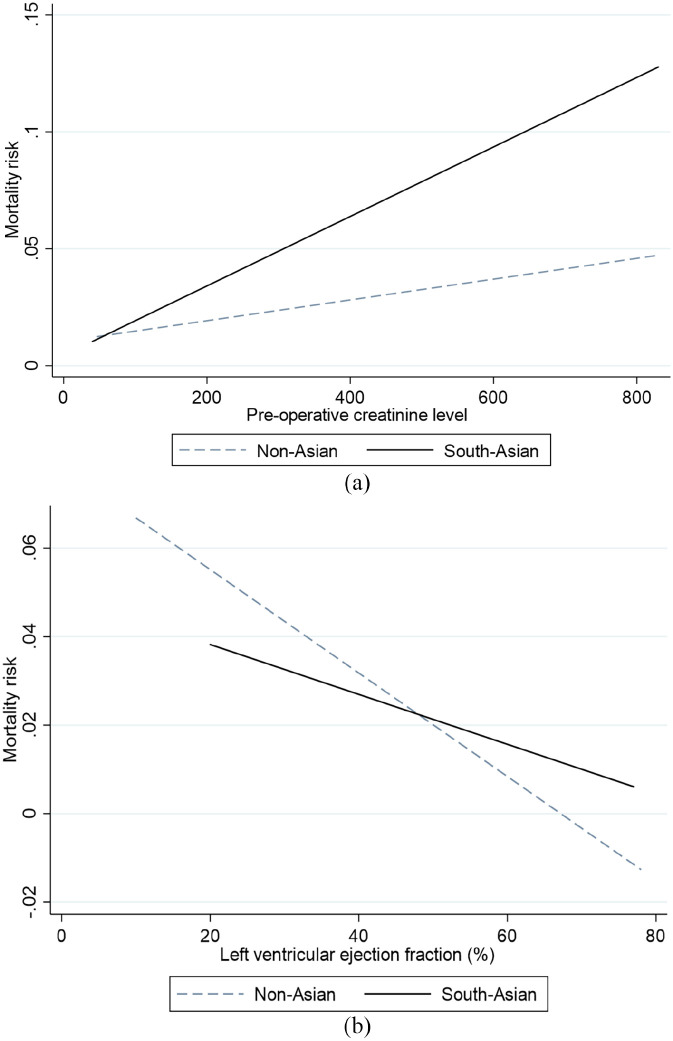
Best-fit plots: (a) Logistic regression analysis of the impact of pre-operative serum creatinine on mortality risk. In the South-Asian cohort (solid line) the association is significant (logistic regression, odds ratio 1.10, 95% CI 1.04–1.07, p = 0.015), whilst the influence of creatinine on mortality was absent in non-Asian patients (dashed line) (p = 0.492) and (b) logistic regression analysis of the impact of pre-operative left ventricular ejection fraction on mortality risk. In the non-Asian cohort (dashed line) the association between ventricular impairment and mortality is significant (logistic regression odds ratio 5.41, 95% CI 1.51–19.45, p = 0.01), whilst this had no influence in the South-Asian patients (solid line) (p = 0.385).

Further subgroup analysis with (the four aforementioned) predictive covariates was undertaken, to ascertain their influence in either of the groups. A significant association between the pre-operative creatinine levels and mortality was found in the South-Asian population (OR 1.10, 95% CI 1.04–1.07, p = 0.015), whilst the influence of creatinine on mortality was absent in non-Asian patients (p = 0.492). Figure 1 shows the distribution of pre-operative serum creatinine levels in patients that died between both cohorts. Figure 2(a) displays the trend between operative mortality risk and creatinine levels, highlighting the difference between non-Asian and South-Asian cohorts.

In the non-Asian cohort, pre-operative LV impairment remained a strong predictor of mortality (OR 5.41, 95% CI 1.51–19.45, p = 0.01), whilst this had no influence in the South-Asian patients (p = 0.385). Figure 2(b) displays the trend between pre-operative ventricular function and the mortality risk, highlighting the difference between non-Asian and South-Asian cohorts. Age remained a predictor of mortality in non-Asian (p = 0.031) and South-Asian (p = 0.002) cohorts.

### Composite complications

A composite value of complication, incorporating operative mortality, stroke, re-operation, hemofiltration, and pneumonia was formulated. Using a multivariate model with ordinal logistic regression analysis (Table 3), the primary predictors of short-term complications were found to be age (coefficient 0.034, 95% CI 0.012–0.057, p = 0.002) and creatinine (coefficient 0.002, 95% CI 0.0004–0.004, p = 0.019). Other patient factors such as gender, smoking history, diabetes, and hypertension were found not to significantly affect the composite complication outcome.

**Table 3.** Multivariate regression analysis, predictors of composite complication.

Covariate	Coefficient	95% CI	Standard error	p-Value
Whole cohort				
Age	0.034	0.012–0.057	0.011	**0.002**
South-Asian ethnicity	−0.128	0.59–0.33	0.24	0.585
Smoking	0.271	0.082–0.623	0.18	0.132
Creatinine	0.0024	0.0003–0.0045	0.001	**0.019**
Dyspnea	0.137	0.173–0.447	0.16	0.387
LV impairment	0.0026	0.025–0.03	0.014	0.855
Cross clamp time	0.0064	0.001–0.014	0.004	0.089
South-Asian cohort only				
Age	0.043	0.016–0.71	0.014	**0.002**
Creatinine	0.0011	−0.0011 to 0.0034	0.0012	0.330
Non-Asian cohort only				
Age	0.016	−0.91 to 0.042	0.013	0.208
Creatinine	0.0016	−0.0012 to 0.0044	0.0014	0.270

Results are from ordinal logistic regression.Bold type denotes statistical significance

**Figure 3.**
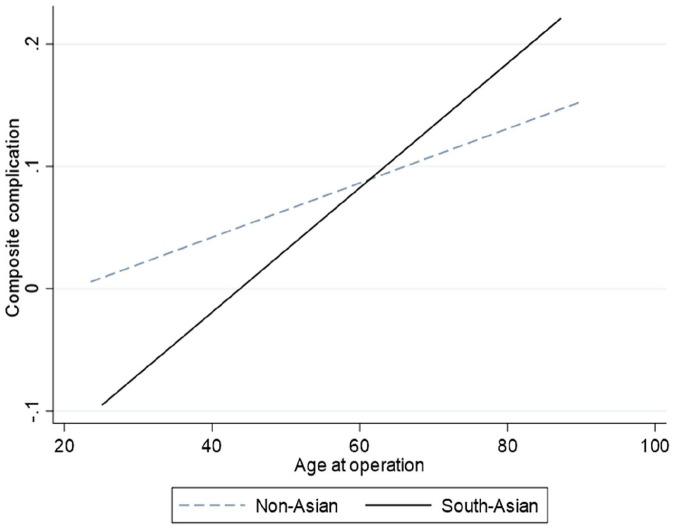
The impact of age on the risk of short term (composite) complications following coronary artery bypass graft surgery. In the South-Asian cohort (solid line) the association between age and short-term complications is significant (ordinal logistic regression coefficient 0.043, 95% CI 0.016–0.71, p = 0.002) whilst this had no influence in the non-Asian patients (p = 0.208).

Subgroup analysis found age to be a significant predictor of worse composite complication (coefficient 0.043, 95% CI 0.016–0.71, p = 0.002) whilst age had no influence in the non-Asian cohort (p = 0.208). The difference in these trends is displayed in Figure 3.

### Survival analysis

**Figure 4.**
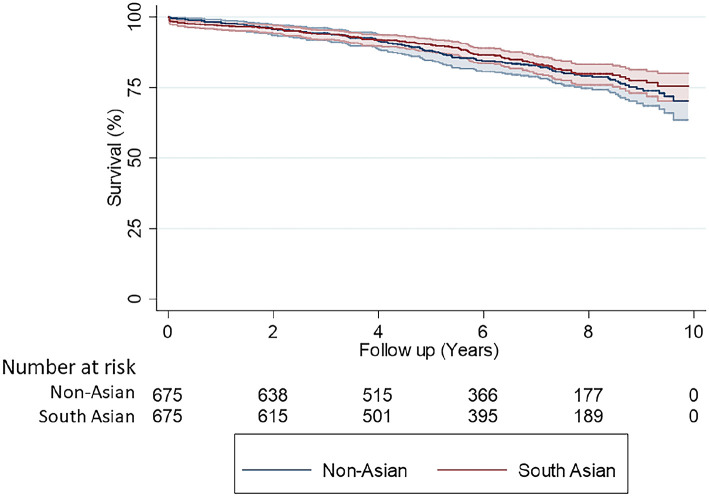
Kaplan-Meier survival analysis comparing South-Asian patients with non-Asian patients (propensity matched cohorts *n* = 675 vs 675).

Survival data was available for up to 10 years for both cohorts (total follow-up time 7801 patient years). Mean follow up time was 6.1 ± 2.7 years. Kaplan Meier analysis showed similar 10-year survival between South-Asian (83.2%) and non-Asian (85.9%) patients, logrank test: p = 0.417 (Figure 4).

Cox-proportional hazards model analysis found raised serum creatinine to be a significant predictor of worse survival (hazard ratio (HR) 1.004, 95% CI 1.003–1.005, p < 0.001) (Table 4). Similarly, advanced age (HR 1.055, 95% CI 1.038–1.073, p < 0.001) and reduced LVEF (HR 0.980, 95% CI 0.963–0.996, p = 0.018) were significant predictors of worse long-term survival. According to the model, ethnicity was not a predictor (p = 0.371), neither were NYHA, diabetes, or gender (p > 0.05).

**Table 4.** Cox regression analysis, influence of pre-operative co-variates on long-term survival post CABG surgery.

Covariate	Hazard ratio	95% CI	Standard error	p-Value
Age	1.055	1.038–1.073	0.009	**<0.001**
South-Asian ethnicity	0.865	0.630–1.188	0.140	0.371
Creatinine	1.004	1.003–1.005	0.005	**<0.001**
LVEF	0.980	0.963–0.996	0.008	**0.018**
Gender	0.928	0.642–1.341	0.174	0.691
Diabetes	1.348	0.971–1.971	0.226	0.074
NYHA class	1.149	0.954–1.384	0.109	0.143

Bold type denotes statistical significance.

## Discussion

The impact of ethnic variations in patients undergoing cardiac surgery may have important implications for predicting adverse outcomes and risk-stratification. In the present analysis, whilst the operative mortality between both groups were similar, the predictors of mortality in the different ethnic cohorts varied significantly, highlighting the importance of a more nuanced approach to pre-operative panning for patients of varying ethnicities. Despite, these differences in short-term outcome, survivorship in the long-term was not different between the different ethnic groups.

### Variation in ethnic response to CABG surgery

A number of studies have explored the difference in outcomes amongst patients of varying ethnic backgrounds with contrasting conclusions.^12,13^ However, in a recent meta-analysis, a large pooled comparison between Asian (*n* = 27,820) and Caucasian patients (*n* = 1,081,642) found in-hospital mortality to be significantly higher in Asian patients (OR 1.33, 95% CI 1.05–1.69, p = 0.02).^5^ The study also found a higher mortality rate in Black patients undergoing CABG when compared to Caucasian patients, whilst no significant difference was identified when compared to patients of a Hispanic background.

Variations in metabolic signature between different population subgroups have long been the subject of interest amongst clinicians in appropriating correct therapy for cardiovascular disease, including heart failure,^14^ atherosclerosis,^15^ and hypertension.^16^ There is evidence for wide variations in enzyme isoforms and cellular receptors lead to ethnic diversity in drug response and pharmacokinetics, with indicating a strong underlying genetic component to the observed biodiversity.

Metabolic syndrome is defined as a constellation of metabolic disturbances including; insulin resistance, central adiposity, dyslipidemia, and hypertension to name a few.^17,18^ This can be hypothesized to account for such differences between South-Asian population and the rest of the population. It has been shown that those with components of metabolic syndrome are twice as likely to have coronary heart disease than those without. Indeed, the prevalence of the metabolic syndrome has been reported higher in South-Asian patients compared to other ethnicities.^17^ This may underly a pathological determinant of coronary heart disease pertinent to this ethnic group which could potentially influence disease trajectory, response to treatment, and recovery from surgery.

### South-Asian ethnicity and renal function

Renal function is a well-known independent risk factor in patients undergoing CABG and its measure can indicate the likelihood of all-cause mortality and complications.^19^ A number of markers such as Neutrophil Associated Gelatinase Lipocalin (NGAL) and microalbuminemia have been identified to measure this,^20,21^ but in particular, creatinine clearance has been identified to be a strong predictor of CABG outcomes and prognosis.^22^ Whilst such biomarkers are better markers for kidney function, their use in pre-operative planning for cardiac surgery is limited to measurements of serum creatinine, which in this study has been demonstrated as significant predictor of adverse outcome. Furthermore, post-operative serum creatinine levels have been found to be to be an assessment of global tissue hypoperfusion alongside a predictor of an early acute kidney injury.^23,24^

Cardiopulmonary bypass is a recognized risk factor for post-operative renal dysfunction.^25^ The reduced perfusion pressure and activation of proinflammatory mediators leads to an increase in renal tissue injury.^26^ NGAL, having previously been identified in animal models, is seen to be a predictor of ischemic renal injury and increases in levels are seen before that of serum creatinine.^20^ Furthermore, microalbuminemia is seen to be more sensitive to manifestations of renal injury and is particularly linked to patients with Type 2 Diabetes (18). Shafranksaya et al.^21^ found that determination of microalbuminemia before and after CABG could prove to be a useful predictor of early AKI.

Another important point for consideration in our present study is the selective negative impact of LV impairment on operative mortality in non-Asian patients. LV dysfunction has been well recognized as a predictor of adverse outcome following cardiac surgery and incorporated into widely used risk-stratification tools for many years.^27^ However, this may indicate that, in South-Asian patients, selective risk-factors associated with metabolic disturbances supersede other recognized predictors of adverse outcome, such as pre-operative cardiac dysfunction.

### Strengths and limitations

The unique findings of the present study are powered by a large population size with comparable cohorts via propensity score matching. Our center serves a large diverse population of patients which provided the interesting data for analysis as well as a contemporary cohort, the investigation of which has discovered important correlations that may aid in operative risk stratification. We have also included prospectively collected data in consecutive patients over a set period of time, thus reducing the selection bias involved. Creatinine is a widely used and available biomarker which increases the translational potential of our work.

Despite this, in expert fields, creatinine is not the optimal marker for renal function. Investigations that incorporate more elaborate methods of renal assessment in various ethnic groups and ascertaining this impact on the outcomes from CABG will be important for future studies. Furthermore, future analyses that adjust for finer operative and CPB-related impact on kidney function, such as hematocrit preserved, hemodilution, and goal directed perfusion, will be crucial to better inform these outcomes.

Another limitation is the grouping of patients: South Asian ethnicity encompasses a large number of sub-groups which could have perceived differences in pre-operative variables. Furthermore, additional data on social deprivation status and long-term follow up may better-inform our outcomes.

When transferring our findings to clinical practice, it is also important to note that our cohort did not include other common revascularization strategies, such as multiple arterial conduits or off-pump surgery.

## Conclusion

Pre-operative creatinine levels predict early mortality in CABG amongst the South-Asian population, whilst advanced age are stronger predictors of short-term complications compared to matched non-Asian patients. By repeating this study on a larger scale, further investigation may help identify ethnicity as a valuable screening tool for pre-operative risk stratification.

## Supplemental Material

sj-pdf-1-prf-10.1177_02676591211037577 – Supplemental material for Predictors of outcome after CABG in the South-Asian community: a propensity matched analysis Click here for additional data file. Supplemental material, sj-pdf-1-prf-10.1177_02676591211037577 for Predictors of outcome after CABG in the South-Asian community: a propensity matched analysis by Mohammad Yousuf Salmasi, Ramanish Ravishankar, Yusuf Abdullahi, Philip Hartley, Panagiotis G Kyriazis, Thanos Athanasiou and Prakash Punjabi in Perfusion
