# Supplementary material for: Predictors of outcome after CABG in the South-Asian community: a propensity matched analysis
Source: Perfusion. 2021 Aug 7;38(1):75–84. doi: 10.1177/02676591211037577 (PMC9841816; doi:10.1177/02676591211037577)
Supplement: sj-pdf-1-prf-10.1177_02676591211037577 – Supplemental material for Predictors of outcome after CABG in the South-Asian community: a propensity matched analysis [file sj-pdf-1-prf-10.1177_02676591211037577.pdf]

Appendix

Baseline variables and operative time for Non-Asian and South-Asian patients

Covariate	Non-Asian (n=1158)	South Asian (n=799)	P-value
Age	63.0 ± 10.7	66.3 ± 10.7	**<0.001**
Female	278 (24.0%)	172 (21.5%)	0.481
NYHA class	2.3 ± 0.2	2.3 ± 0.3	0.622
NYHA III - IV	398 (34.4%)	293 (36.7%)	
Diabetes	387 (33.4%)	492 (61.5%)	**<0.001**
Hypertension	1021 (88.2%)	747 (93.5%)	0.120
Smoking history	786 (67.9%)	427 (53.4%)	**<0.001**
Creatinine	95.9 ± 69.3	106.6 ± 92.0	0.998
Renal failure (stage IV-V)	26 (2.2%)	32 (4.0%)	**0.022**
COPD	108 (9.3%)	55 (6.9%)	**0.049**
Neurological dysfunction	33	19	0.530
Peripheral vascular disease	87	58	0.834
Extent of coronary disease			**<0.001**
1	170	71	
2	191	132	
3 or more	797	569	
LVEF	53.1 ± 8.2	52.7 ± 7.8	0.204
LV impairment	265	177	
Height	169.9 ± 9.5	166.4 ± 12.0	
Weight	81.0 ± 16.2	77.2 ± 15.1	
BMI	27.9 ± 4.8	27.7 ± 4.9	0.280
Cross clamp time	50.6 ± 34.0	42.1 ± 24.6	
CPB time	87.6 ± 51.1	78.6 ± 33.1	

LVEF: left ventricular ejection fraction; COPD: chronic obstructive pulmonary disease; BMI: body mass index; NYHA: New York Heart Association; CPB: cardiopulmonary bypass
